# Understanding therapists’ perceived determinants of trauma narrative use

**DOI:** 10.1186/s43058-021-00231-9

**Published:** 2021-12-02

**Authors:** Hannah E. Frank, Briana S. Last, Reem AlRabiah, Jessica Fishman, Brittany N. Rudd, Hilary E. Kratz, Colleen Harker, Sara Fernandez-Marcote, Kamilah Jackson, Carrie Comeau, Sosunmolu Shoyinka, Rinad S. Beidas

**Affiliations:** 1 Department of Psychology, Temple University, Philadelphia, PA USA; 2 Department of Psychiatry and Human Behavior, Brown University, Providence, RI USA; 3 Department of Psychology, University of Pennsylvania, Philadelphia, PA USA; 4 Department of Psychiatry, University of Pennsylvania Perelman School of Medicine, Philadelphia, PA USA; 5 Annenberg School for Communication, University of Pennsylvania, Philadelphia, PA USA; 6 Penn Implementation Science Center at the Leonard Davis Institute of Health Economics (PISCE@LDI), University of Pennsylvania, Philadelphia, PA USA; 7 Department of Psychiatry, University of Illinois at Chicago College of Medicine, Chicago, IL USA; 8 Department of Psychology, La Salle University, Philadelphia, PA USA; 9 Community Behavioral Health, Philadelphia, PA USA; 10 Department of Behavioral Health and Intellectual Disability Services, Philadelphia, PA USA; 11 PerformCare New Jersey, Robbinsville, NJ USA; 12 Department of Medical Ethics and Health Policy, University of Pennsylvania Perelman School of Medicine, Philadelphia, PA USA; 13 Department of Medicine, University of Pennsylvania Perelman School of Medicine, Philadelphia, PA USA; 14 Penn Medicine Nudge Unit, University of Pennsylvania Health System, Philadelphia, PA USA

**Keywords:** Implementation science, Determinants, Trauma-focused cognitive behavioral therapy, Theory of Planned Behavior, Consolidated Framework for Implementation Research

## Abstract

**Background:**

Trauma narratives are a critical, exposure-based component of trauma-focused cognitive-behavioral therapy, yet community therapists rarely use them. Given evidence that intentions to deliver elements of cognitive behavioral therapy vary by component, and that intentions to deliver exposure are the weakest, this study focused specifically on trauma narratives. We drew on a social psychology causal theory (Theory of Planned Behavior (TPB)) and an implementation science framework (the Consolidated Framework for Implementation Research (CFIR)) to glean insight into multilevel influences on trauma narrative use. While the CFIR offers a broad list of factors potentially affecting implementation, the TPB offers causal pathways between individual-level constructs that predict behavior, including the uptake of an evidence-based intervention. The integration of these approaches may provide a more complete understanding of factors affecting therapists’ use of TNs.

**Methods:**

Therapists (*n=*65) trained in trauma-focused cognitive behavioral therapy completed a survey about their use of and beliefs about trauma narratives. Content analysis was used to identify common beliefs about trauma narratives. A subset of participants (*n=*17) completed follow-up qualitative interviews, which were analyzed using an integrated approach informed by the CFIR.

**Results:**

While most participants reported high intentions to use TNs, nearly half reported that they did not use TNs in the last 6 months. Survey data indicate a number of TPB-related determinants related to using trauma narratives. Qualitative interviews identified CFIR-relevant contextual factors that may influence constructs central to TPB.

**Conclusions:**

These results highlight the importance of integrating approaches that address multiple theoretical determinants of therapist behavior, including therapist, organizational, and client factors with causal explanations to explain implementation behavior.

Contributions to the literature

- Trauma narratives are a key part of treatment for children who experience trauma, but they are rarely used in clinical practice
- This study used quantitative and qualitative methods to assess community therapists’ perceptions of trauma narratives
- Several factors, such as agency support and family engagement in treatment may affect therapists’ trauma narrative use
- Combining a causal theory (the Theory of Planned Behavior) and an implementation science framework (the Consolidated Framework for Implementation Research) may provide a more complete understanding of barriers to implementation

## Background

Although evidence-based interventions (EBIs) exist for youth mental health, these interventions are rarely used in usual care settings [1]. Research focused on improving the implementation of youth mental health EBIs has identified barriers and facilitators to implementation [2, 3]. However, even when clinicians increase their use of EBIs, some components are more likely to be used than others. Evidence suggests that “active ingredients,” or the most effective components of EBIs, are often delivered less frequently [4], likely reducing the effectiveness of interventions in usual care settings. Increasing clinician use of such intervention components necessitates an in-depth understanding of implementation determinants, as well as identification of potential causal pathways that predict their use [5].

One intervention component that is used the *least* frequently despite having the strongest evidence for its effectiveness is exposure, which involves approaching feared situations in a gradual and supportive manner [6, 7]. Even among clinicians trained in cognitive behavioral interventions for mental health disorders, less than a quarter report using exposure [8], which is a key treatment component for anxiety-based disorders including post-traumatic stress disorder (PTSD). Providing quality care for PTSD and persistent trauma symptoms is a priority given that the majority of youth in the United States experience traumatic events before adulthood, [9]. The delivery of effective treatment reduces the negative sequelae associated with untreated trauma symptoms, including suicidal thoughts and behaviors [10] and high medical costs [11]. Trauma-focused cognitive behavioral therapy (TF-CBT) is one evidence-based treatment for youth experiencing PTSD [12, 13] that has demonstrated effectiveness in reducing symptoms [14, 15]. A core component of TF-CBT is the trauma narrative (TN), a type of exposure in which youth are encouraged to gradually increase contact with details of the trauma while “unlink[ing] thoughts, reminders, or discussions of the traumatic event from overwhelming negative emotions” (12 , p. 172). TNs provide insight into specific dysfunctional trauma-related beliefs that can be addressed and corrected in treatment [16].

Much like other exposure-based intervention components, TNs are rarely used in practice. Studies examining therapists’ perspectives of TF-CBT found that TNs were rated as one of the most difficult TF-CBT components to implement and considered to be only appropriate for some children [17, 18]. One study found that, although all evidence-based protocols for trauma include some element of exposure, only 14–22% of trauma-exposed youth received exposure during treatment [8]. This is consistent with the finding that therapists report being less motivated to deliver exposure compared to other CBT elements [19] and express concerns about its appropriateness for some settings (e.g., schools [18, 20]). This may be due to therapist discomfort, or concern that engaging in the exposure may cause harm [21]. Therapists are likely attuned to the heightened negative affect and physiological arousal that youth report experiencing during the development and processing of the TN [22]. However, youth acknowledge the benefits and functional improvements they experience because of completing TNs [22, 23].

Although several determinants to TN use have been identified, much remains unknown about how these factors influence TN implementation. One validated causal theory that has been adapted from social psychology and increasingly applied to implementation science is the Theory of Planned Behavior (TPB [24–28]). The TPB states that behavioral intentions, which capture one’s strength of motivation to perform a behavior, are the strongest predictor of actual behavior. Intentions are informed by attitudes, subjective norms, and perceived behavioral control (also called self-efficacy). Attitudes are posited to be affected by perceived advantages and disadvantages of performing the behavior [24, 29]. Norms are based on perceptions of what one is expected to do, as well as what one believes other people do. Finally, self-efficacy is influenced by beliefs about one’s ability to perform the behavior. The TPB causal model suggests that understanding the role played by attitudes, norms, and self-efficacy is critical to identifying the mechanisms underlying intention to use TN. The TPB has several strengths, including standardized, specific definitions of constructs that predict individual behaviors, standardized, predictive methods for measuring these constructs, and a causal explanation for factors influencing implementation. Multi-level factors are likely to influence the beliefs underlying intentions and to determine the degree to which intentions are correlated with actual behavior [27, 30, 31]. For example, policies, resources, and intervention characteristics can influence the relationship between intentions to use and actual use of an EBI. Prior research using the TPB has identified that constructs such as therapists’ perceptions of manualized protocols (attitudes), consumer awareness of EBIs (subjective norms), and insufficient time for training in EBIs (perceived behavior control) are barriers to EBI use [32].

The Consolidated Framework for Implementation Research (CFIR [33]) is a leading implementation determinant framework that explicitly focuses on the importance of multi-level influences on implementation outcomes [34]. The CFIR includes five general domains: intervention characteristics (i.e., the features of the intervention), inner setting (i.e., organizational culture and climate), outer setting (i.e., the political and social climate), characteristics of individuals (e.g., therapists’ knowledge and beliefs), and implementation process (e.g., planning, engagement). The CFIR includes comprehensive terminology for each domain but does not identify any causal mechanisms or provide insight into *how* or *why* change takes place [35]. Furthermore, the CFIR includes fewer specifics compared to the TPB on therapist-level determinants [36]. When used together, the CFIR and the TPB may ideally complement each other and provide a more thorough understanding of factors influencing EBI implementation.

The present study examined therapist perspectives of using TNs in community mental health settings after receiving training in TF-CBT within a public mental health system committed to EBI implementation. The first phase of this study identified beliefs that, according to a causal theory (TPB), can influence therapists’ intentions to use TNs. The second phase involved in-depth qualitative interviews with a subset of participants to identify CFIR factors that may influence individuals’ beliefs and TN use. The primary aim of this study was to use mixed methods to integrate insights from a causal theory (TPB) and a determinant framework (CFIR) to enhance our understanding of TN implementation.

## Methods

### Setting

This study took place in Philadelphia, a diverse city of over 1.5 million residents [37]. Public behavioral health services, including mental health and substance use treatment, are funded by Medicaid and managed by Community Behavioral Health (CBH). CBH is a non-profit managed care organization that is part of the larger organization overseeing public behavioral health service delivery in Philadelphia County (the Department of Behavioral Health and Intellectual disAbility Services; DBHIDS). In response to high rates of trauma exposure among youth in Philadelphia, DBHIDS began developing a comprehensive trauma-informed public behavioral health system in 2011. DBHIDS was awarded a National Child Traumatic Stress Initiative Community Treatment and Service Center grant (Category III) from the Substance Abuse and Mental Health Services Administration (SAMHSA) in 2012 and again in 2017 to support the Philadelphia Alliance for Child Trauma Services (PACTS). The goal of the ongoing PACTS initiative is to increase the number of children in Philadelphia receiving evidence-based trauma treatment [38]. Since 2012, PACTS has trained ten cohorts of therapists in TF-CBT with a 2-day workshop and 8 months of bi-weekly consultation calls with a TF-CBT certified master trainer. Training was offered by the TF-CBT national training team and was the same across all cohorts. All PACTS-trained therapists continue to receive support and technical assistance from the PACTS initiative after training, including an annual booster training [38].

### Participants

Participants were therapists (*N*=65) trained through the PACTS initiative. To ensure representativeness across stages of training, this study included participants from all training cohorts. A subset of therapist participants (*n*=17) who completed the initial survey informed by the TPB (Phase 1) was selected for in-depth qualitative interviews informed by the CFIR (Phase 2). All procedures were approved by the City of Philadelphia and the University of Pennsylvania Institutional Review Boards.

### Phase 1

#### Measures

##### Demographics

Questions in the initial survey asked about participants’ age, racial/ethnic background, mental health licensure status, education, occupational title, experience conducting psychotherapy, and PACTS training year.

##### TN intentions and use

Participants rated the strength of their intentions to use TNs using two items that included Likert-scale response options [27]. The first item asked respondents to rate how strongly they agreed/disagreed with the statement, “In the next six months, I intend to use TN with the majority of my patients who receive TF-CBT.” The second item asked respondents to estimate their likelihood of using TNs with the majority of TF-CBT clients in the next 6 months. Participants were also asked about the percentage of TF-CBT clients with whom they had used TNs in the last 6 months.

##### Beliefs about using TN

TPB beliefs were assessed with a belief elicitation survey, which uses standardized procedures to assess the beliefs that underlie intentions [39–41]. The methodology requires representative respondents to identify the “tip of the tongue” beliefs that spontaneously come to mind when they think about the behavior of interest [42]. Rather than relying on preconceived lists of statements identified by researchers, this method identifies stakeholder perspectives more directly. The survey consisted of seven open-ended questions pertaining to therapists’ opinions of using TNs with the majority of their clients. Participants were asked about factors affecting their use of TN with the majority of their patients receiving TF-CBT. Questions assessed their beliefs underlying (a) *attitudes* by asking about advantages and disadvantages of using TNs; (b) *norms* by asking who would disapprove and who would approve of using TNs; and (c) *self-efficacy* by asking what would make it difficult and what would make it easier to use TNs.

#### Procedures

In April and May 2018, PACTS participants who had completed initial training were asked to complete a 10–15-min online survey that asked about demographics, TN intentions and use, and beliefs about using TNs. Prior to completing the survey, therapists provided written or e-consent for all study procedures. Therapists who completed the survey were entered into a lottery to be paid $50 for completing the survey; five participants were randomly selected to be paid. Although the exact number of therapists who received the request to participate in the study is unknown, there are approximately 200 therapists who would have been eligible for the study [43]. Eighty-four therapists opened the survey, and 65 therapists completed the consent process and responded to the questionnaire.

### Phase 2

#### Measures

##### CFIR qualitative interviews

Semi-structured, CFIR-informed qualitative interviews were conducted with participants and focused on therapist beliefs about using TNs^1^. In addition to prompts related to CFIR constructs, participants were asked to elaborate on specific responses they gave during the TPB belief elicitation. Each participant completed one interview lasting 30–60 min. Interviews were audio-recorded and conducted individually at the participants’ office or by phone. Interviews included open-ended questions about therapists’ beliefs about TNs. No further contact was made with participants after the Phase 2 interviews, and they did not provide feedback on transcripts or results. Interviews were conducted by female doctoral (HEF, BSL) and postdoctoral (BNR) trainees with experience in implementation research. The interviewers did not have prior relationships with any of the participants. The only personal information shared about the interviewers was that they were affiliated with the PACTS initiative and interested in understanding therapists’ perceptions of TNs.

#### Procedures

In-depth semi-structured CFIR-informed qualitative interviews were conducted with a subset of participants who responded to the initial survey. Recruitment methods for the qualitative interviews were informed by the Dillman Total Design Survey Method [44] and included two follow-up emails sent one week after the initial email requesting participation in the qualitative interviews. We used purposive sampling to recruit therapists in three groups, including (a) therapists with strong intentions to use TNs who reported using TNs with all or most of their clients (*n*=5), (b) therapists who reported weaker intentions to use TNs and who had mid-levels of past TN use (*n*=4), and (c) therapists with strong intentions to use TNs, but who had used TNs with none or few clients (*n*=8). Of the 26 participants invited to complete the qualitative interview, 17 participants completed interviews by phone or in person (65%). Of note, all participants with weak intentions (*N*=4) agreed to participate. Those who declined either did not respond to attempts to contact them (*n=*9) or reported insufficient time available to participate (*n*=3). Participants received $50 for completing the interviews.

### Data management and analysis

#### TPB belief elicitation (Phase 1)

Content analysis was done collaboratively by two doctoral students (HEF and BSL) to create categories of similar responses to each question. Disagreements were discussed until consensus was reached. As recommended by prior research [26, 45], we report the categories endorsed by ≥10% of participants. To integrate TPB and CFIR findings, each of the TPB beliefs was categorized as belonging to one or more of the five CFIR domains. as shown in Tables 2, 3, and 4. For instance, the belief that supervisors within the agency would approve of TN use was labeled as being relevant to the CFIR Inner Setting domain. Differences between “implementers” (those who used TNs) and “non-implementers” (those who did not use TNs) were identified by visually comparing the number of responses for each belief.

#### CFIR Qualitative Interviews (Phase 2)

Qualitative interviews were transcribed by undergraduate research assistants (RAs) and analyzed using an integrated analysis [46], identifying a priori attributes (CFIR constructs) and also using modified grounded theory [47, 48]. Initial codes included a priori codes specific to the CFIR (i.e., intervention characteristics, inner setting, outer setting, characteristics of individuals, implementation process), which were not mutually exclusive. Statements were also coded as being a barrier or a facilitator based on therapists’ descriptions of how each factor affected their TN use. Additional codes, including “family characteristics” and “modifications/adaptations to TNs,” were generated using grounded theory. These codes are considered sub-themes of outer setting and intervention characteristics, respectively.

Based on a review of transcriptions, thematic saturation was reached. Transcripts were coded by two coders, including an advanced doctoral student (HEF) and an undergraduate RA. A subset of transcripts was collaboratively reviewed to develop a codebook and refine code definitions. Then, three transcripts were coded separately to achieve initial reliability (agreement on all codes >90%). Both coders coded the remainder of transcripts independently, overlapping on a subset of transcripts (*n*=6) to assess final reliability for all codes, which remained above 90% agreement (*range* = 90.12–96.73%). Codes were summarized in NVivo and examined for themes. Frequencies for each code were not calculated given that qualitative research does not rely on counting or quantifying responses [49, 50]. A post hoc mixed methods analysis was conducted to determine differences in themes between each of the three sampled groups. Responses for each code were summarized for participants in each subgroup. Review and discussion of subgroup summaries was completed to identify whether there were any meaningful differences between groups. Given the small population of the low intentions subgroup (*n*=4), saturation of themes was not achieved for this subgroup on any code.

## Results

### Demographic characteristics

Demographic characteristics for Phase 1 and Phase 2 participants are shown in Table 1. All therapists worked in community clinics within the CBH network. Most therapists trained in the PACTS initiative work in outpatient settings, but some therapists also work in residential, home-based, and school-based treatment settings. Information about the specific setting(s) in which therapists worked was not systematically collected.

**Table 1:** Demographic characteristics

	Phase 1 (*N*=65)	Phase 2 (*N*=17)
Characteristic	***N*** (%) or mean (SD)	***N*** (%) or mean (SD)
**Age**	34.26 (10.62)	32.24 (9.74)
**Gender (woman)**	60 (92.3%)	17 (100%)
**Hispanic/Latinx**	16 (24.6%)	4 (23.5%)
**Race**		
American Indian/Alaskan Native	1 (1.5%)	0
Asian	4 (6.2%)	0
Black or African American	11 (16.9%)	1 (5.9%)
Native Hawaiian or Pacific Islander	1 (1.5%)	0
White	47 (72.3%)	15 (88.2%)
Other	6 (9.2%)	1 (5.9%)
**Licensed**	34 (52.3%)	11 (64.7%)
**License type**		
Psychologist	2 (3.1%)	0
Professional Counselor	9 (13.8%)	2 (11.8%)
Clinical Social Worker	15 (23.1%)	5 (29.4%)
Licensed Social Worker	7 (10.8%)	3 (17.6%)
Marriage and Family Therapist	1 (1.5%)	1 (5.9%)
Not reported	2 (3.1%)	0
N/A (not licensed)	29 (44.6%)	6 (35.3%)
**Highest degree**		
Master’s	61 (93.8%)	16 (94.1%)
Doctorate	4 (6.2%)	1 (5.9%)
**Completion of PACTS training**		
2012	2 (3.1%)	1 (5.9%)
2014	2 (3.1%)	0
2015	6 (9.2%)	3 (17.6%)
2016	6 (9.2%)	2 (11.8%)
2017	27 (41.5%)	4 (23.5%)
2018 (year study was conducted)	19 (29.2%)	6 (35.3%)
Not reported	3 (4.7%)	1 (5.9%)

### TN intentions and use

Therapists agreed with the statement that “I intend to” use TNs with the majority of patients who receive TF-CBT (*M=*4.22, SD = 1.03) on a 5-point scale. They also reported that they were “likely to use” TNs (*M*=5.94, SD =1.18) on a 7-point scale. However, 47.7% of therapists reported that they did not use TNs with patients receiving TF-CBT over the last 6 months. Recency of training did not explain this finding, as being trained in the past year versus in prior years did not predict TN use, *c*^2^(4, *N*=61)=6.15, *p*=.19. Participants who completed qualitative interviews reported a higher likelihood of using TNs in the next 6 months (*M*=6.65, SD=0.79) than participants who did not complete qualitative interviews (*M*=5.68, SD=1.20), *t*(43.57) = − 3.73, *p*=.001; no other differences were found between participants who did and did not complete qualitative interviews.

### Phase 1: TPB belief elicitation

Beliefs are described below, along with the CFIR domains they reference (in italics). First, respondents were asked about the advantages of using TNs (i.e., beliefs underlying attitudes), as shown in Table 2. Some responses were consistent with what CFIR would categorize as *outer setting* (which includes family characteristics), such as involving parents in a supportive role (12% of respondents). In addition, therapists identified positive *intervention characteristics*, such as the value of using TNs to help clients better understand their trauma and gain mastery over it (14% of respondents). When asked to share the disadvantages of using TNs, many participants (25%) stated that there were none. Those who did identify disadvantages noted concerns related to *outer setting,* including caregivers being unprepared, unhelpful, or unsupportive (17% of respondents). Some therapists also identified concerns related to *intervention characteristics*, including that TNs would worsen the client’s symptoms (15% of respondents). The same beliefs emerged for both implementers and non-implementers. However, implementers more frequently mentioned a reduction in cognitive distortions, the benefits of gradual exposures, and parents providing support as advantages. Non-implementers more frequently endorsed concerns about worsening symptoms as a disadvantage.

**Table 2:** Phase 1: Beliefs underlying attitudes about using trauma narratives with majority of clients receiving TF-CBT and CFIR domains

*N* = 65	Count	%	CFIR domain
Perceived **advantages** of using TN			
Child is better able to process their trauma	22	34	Int
Child learns to identify and address cognitive distortions	15	23	Int
Reduction in trauma symptoms	10	15	Int
Allows for gradual exposure to trauma	10	15	Int
Client and therapist gain sense of mastery/empowerment over trauma	9	14	Int
Parents able to provide support	8	12	OS
Reduction in child avoidance	7	11	Int
Perceived **disadvantages** of using TN			
None	16	25	--
Negative caregiver reactions	11	17	OS
Increase/worsening of client symptoms	10	15	Int

*Notes*: Count and percentage refer to the number of participants who endorsed each answer. Percentages may sum to more than 100% because participants could list multiple answers. All beliefs are inclusive to the “individuals involved” CFIR domain. We also mapped these beliefs onto other potential CFIR domains with which they are associated.

*Abbreviations*: *Int* intervention (i.e., features of intervention), *IS* inner setting (i.e., organizational culture and climate), *OS* outer setting (i.e., political and social climate; includes *family characteristics*); *Ind* characteristics of individuals (i.e., therapists’ knowledge and beliefs), *IP* implementation process (e.g., planning, engagement)

Responses regarding who would approve and disapprove of TN use (to identify normative beliefs) are shown in Table 3. Participants reported that those who would approve included supervisors (46% of respondents; classified by CFIR as part of the *inner setting*), as well as caregivers (31% of respondents) and clients (22% of respondents; classified by CFIR as *outer setting*). Several participants commented on the importance of agency-level support more broadly (15% of respondents). Many participants (45%) indicated that no one would disapprove of using TNs. However, others reported that caregivers (28% of respondents) and/or clients (15% of respondents) might disapprove. Compared to implementers, non-implementers more frequently endorsed concerns that clients might disapprove of TN use.

**Table 3:** Phase 1: Normative beliefs about using trauma narratives with majority of clients receiving TF-CBT and the CFIR domains

*N* = 65	Count	%	CFIR domain
Those who are perceived to **approve** of the therapist using TN			
Supervisor	30	46	IS
Caregiver	20	31	OS
Client	14	22	OS
Agency	10	15	IS
Those who are perceived to **disapprove** of the therapist using TN			
No one	29	45	--
Caregiver	18	28	OS
Client	10	15	OS

*Notes*: Count and percentage refer to the number of participants who endorsed each answer. Percentages may sum to more than 100% because participants could list multiple answers. All beliefs are inclusive to the “individuals involved” CFIR domain. We also mapped these beliefs onto other potential CFIR domains with which they are associated.

*Abbreviations*: *Int* intervention (i.e., features of intervention), *IS* inner setting (i.e., organizational culture and climate), *OS* outer setting (i.e., political and social climate; includes *family characteristics*), *Ind* characteristics of individuals (i.e., therapists’ knowledge and beliefs), *IP* implementation process (e.g., planning, engagement)

Finally, self-efficacy beliefs were assessed with prompts about what would make it easier to use TNs (Table 4). Therapists stated that support from caregivers (23% of respondents) and consistent client attendance (20% of respondents) would make it easier (*outer setting*). Participants also identified *implementation process* factors (e.g., training and supervision) as making it easier to use TNs (14% of respondents). Topics identified as making TN use difficult largely involved client factors, such as lack of caregiver support (26% of respondents), client avoidance (17% of respondents), and clients not being ready (11% of respondents). Compared to non-implementers, implementers more frequently noted concerns about client readiness for TNs.

**Table 4:** Phase 1: Self-efficacy (and perceived behavioral control) beliefs about using trauma narratives with majority of clients receiving TF-CBT and CFIR domains

*N* = 65	Count	%	CFIR domain
What would make it **easier** to use TN			
Caregiver support	15	23	OS
Client attendance	13	20	OS
More training/supervision	9	14	IP
What makes it **difficult** to use TN			
Inconsistent attendance/treatment dropout	18	28	OS
Lack of caregiver involvement/support	17	26	OS
Client avoidance	11	17	OS
Child is too young	7	11	OS
Client resists/refuses	7	11	OS
Client is not ready	7	11	OS

*Notes*: Count and percentage refer to the number of participants who endorsed each answer. Percentages may sum to more than 100% because participants could list multiple answers. All beliefs are inclusive to the “individuals involved” CFIR domain. We also mapped these beliefs onto other potential CFIR domains with which they are associated.

*Abbreviations*: *Int* intervention (i.e., features of intervention), *IS* inner setting (i.e., organizational culture and climate), *OS* outer setting (i.e., political and social climate; includes *Family Characteristics*), *Ind* characteristics of individuals (i.e., therapists’ knowledge and beliefs), *IP* implementation process (e.g., planning, engagement)

### Phase 2: CFIR qualitative interviews

Responses for the a priori CFIR codes and grounded theory subcodes are summarized below. Table 5 includes example quotes for each code.

**Table 5:** Phase 2: Example quotes for each code

Code	Example quote
**Intervention characteristics**	“I really like how structured it is. I think that’s very helpful, having the chapters and you know, but also having some freedom to let the kid choose, like okay which chapter are we going to work on today? That’s been helpful to have structure and then also flexibility.”
	“[In] some cases the structure is very helpful for a kid to verbalize what they learn[ed] and how things are different … I’ve seen kids really proud of their narratives and they share with their parent and they’re able to have these “aha” moments.”
***Modifications and adaptations to TNs***	“I tend to do written narratives. I have clients who have done … more of a rap narrative. [They were able to] talk about their trauma history [in a way] that they wouldn’t have been if we did a straight narrative like chapter one, chapter two, chapter three.”
	“I had him dictate it to me because I knew he would really be finicky with grammar and spelling and all of that, and I think that would have held him up a bit.”
**Inner setting**	“[Something that helps is … ] my supervisor and my coworkers being trained also. To have them to immediately bounce [ideas off of].”
**Outer setting**	“I think that when you’re in a situation where the community you’re living in feels really unsafe, it’s hard to get that stuff under control.”
	“I find that the kids that don’t have as many outside stressors happening can do their trauma narratives fairly quickly, whereas the kids that come in and they’re, you know, still going to court and going through that process… that makes it difficult too.”
***Family characteristics***	“I think those cultural pieces sometimes can be really difficult. In certain communities when trauma occurs, you don’t talk about it, you bottle it up and it just goes unsaid…. I asked what happened next in terms of processing it, and she said, ‘Well everything just kind of went on like nothing happened, we didn’t talk about it and we just swept it under the rug as a family.’”
	“It can be difficult when there’s really complex trauma histories … It’s [easier] when there’s a single episode or it’s a same type of abuse that they experience multiple times. But, when it’s physical, emotional, sexual, and neglect and a lot of it, the abuse that impacted them the most might actually be [happening] now.”
**Characteristics of individuals**	“The trauma narrative is honestly one of my favorite parts of TF-CBT cause it’s the culmination of all the work that we’ve already done. We’ve done all the gradual exposure and … they’ve learned all these skills so then being able to actually sit down and write it is really cool to hear… I feel like the gradual exposure has worked on me too and I’m desensitized to their trauma as well.”
	“I remember feeling a little apprehensive beforehand. Is this going to go well? Is the parent going to react in a way that’s going to be helpful for the kid? I remember feeling excited that we were in this moment, but then also just apprehensive and a little bit nervous… And then afterward, feeling relieved and also…very hopeful, and having a sense of completion”
**Implementation process**	“The second booster was really, really helpful for me after seeing clients for many months… I wish there was more work on processing the narratives and more explanations on the narrative because some of the people had different ideas of how long it should be or how thorough it should be or what it should look like. It was helpful in my call when the facilitator read some of their narratives of what they had done.”

#### Intervention characteristics

Therapists generally perceived TF-CBT and TNs positively, often describing them as helpful and effective. There were mixed perspectives regarding whether TNs are sufficiently structured, or whether they are too vague to know exactly how and when to implement each component. For instance, one participant noted that they did not “want to go in and have an agenda and… be sticking to a script. But as I’ve worked with the model, in some ways I’ve wished for even more prescriptive elements.” Participants described a substantial amount of preparation involved when implementing TNs. One participant stated that “being able to devote the time and attention to prepare for trauma narratives can be difficult,” but others said that it becomes easier with experience. Therapists highlighted the difficulty of interruption of services because of non-attendance by the parent and/or child or other emergent life events. The amount of time required to meet with both the parent and child and to cover all necessary topics was also identified as a barrier. However, one participant noted that “even if you’re not spending a whole half of the session doing it… there’s still… an argument for working on it for 15 minutes… to keep that gradual exposure going.” Therapists mentioned the importance of building rapport and a sense of safety with the client prior to beginning TNs and stated that this was made easier by this gradual nature of exposures. There were some concerns about the feasibility of repeatedly reviewing long narratives given time constraints.

#### Modifications and adaptations to TNs

Modifications to TNs were made to address issues of client comorbidity, literacy, attention span, or other life circumstances. Therapists used a variety of mediums for creating TNs, including drawing, rapping, creating PowerPoint presentations, using highlighters, and writing poems. Some participants adjusted the pace of TNs such that they only read parts of a long narrative in response to the child’s needs (e.g., “we went slow for him because he was very guarded and very reserved”) or due to session length. Therapists described various approaches of reading to the child, typing for the child, and getting rid of the TN at the end of treatment. One participant said that they “pick and choose the most impactful chapters [of the TN] that might need more gradual exposure.” The extent to which caregivers were involved in TNs was often adjusted depending on the child’s circumstances. In some cases, it was not feasible for a caregiver to be involved, and some therapists recruited other adults from the child’s life to hear the TN.

#### Inner setting

Therapists described administrative issues as a pervasive barrier affecting TN implementation. Common issues included lack of reimbursement for longer sessions and insufficient time to complete paperwork. Some therapists mentioned that they conduct sessions in communities: “So sometimes you’re meeting in a park or a McDonald’s… so it’s just hard to be discussing it.” There were also concerns about clinic policies related to conducting TNs in the presence of an open legal investigation. Having supervisors and co-workers trained in TF-CBT was reported as a facilitator, whereas being the only trained therapist was a barrier. Participants described agency expectations that TF-CBT would be used as a facilitator. For example, one therapist said, “Where I work, we do employ TF-CBT, that’s kind of what they do there, so I do a trauma narrative with every single kid.” Therapists working in school settings noted consistency in child attendance as a facilitator for TN completion. On the other hand, school-based therapists noted concerns about accessing parents, discontinuing therapy over the summer, and having students return to class dysregulated after completing TNs.

#### Outer setting

One outer setting theme that emerged was a concern about using TNs to treat chronic trauma for clients living in communities with high rates of violence. One participant said, “The population I currently work with is probably one of the hardest ones I’ve ever worked with or tried to implement TF-CBT because they do have so many trauma/potential traumas.” Participants noted that in such cases, some avoidance on the part of the child may be protective. Participants also mentioned external factors, such as court or child protective services involvement, that may hinder the ability to use TNs.

#### Family characteristics

Several key themes emerged related to family factors affecting TN use. Therapists identified poor attendance and dropout as common barriers, whereas caregiver involvement and motivation were common facilitators. Logistics such as transportation and childcare were described as affecting attendance and consistent TN implementation. One participant noted that “sometimes it was just … transportation to get there, even despite concrete resources like [subway] tokens and stuff.” Furthermore, therapists mentioned cultural or family norms about the extent to which speaking about trauma and seeking help to manage it is acceptable. Therapists noted that client characteristics, such as developmental level or time since the trauma occurred, also affected TN delivery. Children who had recently experienced trauma were described by some therapists as not being emotionally ready to start a narrative or too symptomatic to successfully complete one. Therapists reported that it was easier to create TNs with children who were interested in art and stories, whereas more verbal children sometimes had narratives that were too lengthy to repeatedly use.

Therapists reported that a single trauma was easier to treat compared to multiple or ongoing traumas. Participants noted that poor attendance and interruptions in treatment often resulted from financial or housing instability, and required a shift toward focusing on crisis management instead of on trauma. Children in foster care were described as the hardest to treat due to the lack of a consistent caregiver, as well as other sources of instability. For instance, one therapist said that for children living with foster parents, “a lot of the time the households are not very stable or great for them. And they end up having to leave that household because of either their behavior or because they are reenacting trauma stuff… and they end up leaving that household, [which is] logistically really challenging.” Therapists cited good rapport with clients and caregivers as facilitators for completing TNs. For instance, working with parents who are “super engaged and ready, and also [have] a lot of skills in empathy” made it easier to complete TNs. In general, stronger child-caregiver relationships were considered to result in more successful TNs, whereas children who had tense relationships with their caregivers were more difficult to treat.

#### Characteristics of individuals

Therapist characteristics related to TN use included the emotional impact of using TNs. Therapists reported experiencing or worrying about burnout and mitigating this risk by having the support of a team or having variety in the types of cases they treated. Participants also identified a process of desensitization whereby hearing TNs became easier over time. Therapists reported a wide range of emotions they experienced around TNs, including nervous, proud, sad, frustrated, intimidated, and relieved. One therapist described feeling “fulfilled” as a result of “having the child and the parent be able to have the experience [of doing a TN] and bond together over that.” On the other hand, several therapists mentioned doubt and uncertainty when making decisions about how hard to push clients in sharing TNs. One therapist described past instances in which she worried that she may have “pushed a trauma narrative when maybe it wasn’t necessarily needed.”

#### Implementation process

Initial training, booster training, and consultation calls were generally viewed positively. Participants noted a desire for additional materials beyond the treatment manual to support implementation (e.g., slides, handouts), with some indicating that that need was met and others suggesting that more materials would have been useful. The booster training was identified as being critical to support TF-CBT and TN use. Overall, participants felt that training alone was *not* sufficient, and that being able to implement TNs required support via consultation calls (“our call facilitator was giving us lots of examples, which was so helpful”), additional training materials (e.g., “a de-identified narrative”), and adequate support to prepare for sessions (“it can be overwhelming … to prepare for your sessions depending on what’s in the trauma narrative”).

#### Post hoc (mixed methods) analysis

Therapists with high intentions and frequent TN use described using a wider array of mediums (e.g., songs, poems) to implement TNs than other groups. Although all groups described barriers and facilitators related to the inner setting, therapists with high intentions and low TN use reported much more variability in their level of agency support for using TNs compared to other groups. This group also identified more themes related to uncertainty about clients’ readiness for doing TNs. Finally, the group with weaker intentions and moderate TN use did not mention booster sessions or consultation calls, whereas participants in the other groups described them as being integral to implementation success.

#### Proposed causal model

**Fig. 1:**
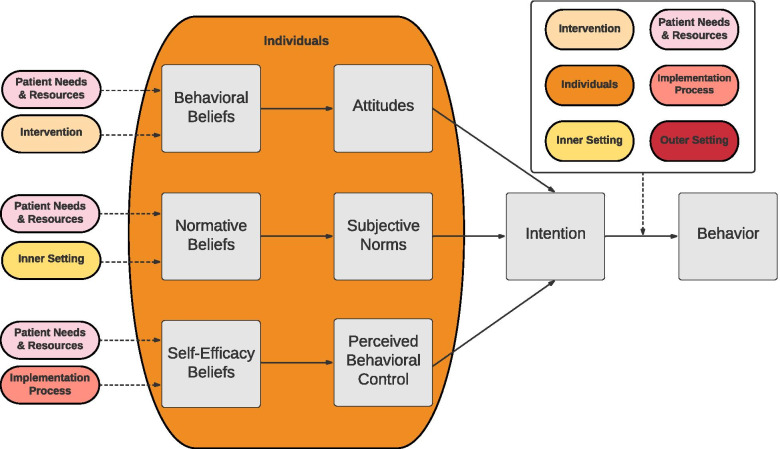
Proposed combined TPB and CFIR model. *Note*: This is a proposed conceptual model that we developed based on our data. Colored ovals represent the CFIR domains and gray boxes represent the TPB. This integrated model may look different for other interventions and contexts

Figure 1 depicts a proposed model that represents our conceptualization of data from the current study. This figure depicts our understanding of how the CFIR and TPB may be combined to provide a causal explanation of behavior in this example. Of note, family characteristics emerged as a distinct theme described as having an impact on TN implementation. This is consistent with research calling for the promotion of “patient needs and resources” to its own CFIR domain [51] and is reflected as such in the figure.

## Discussion

This study integrated a causal theory and a determinant framework to identify factors related to therapists’ TN use in community mental health settings. Most therapists reported strong intentions to use TNs; however, TN use was relatively infrequent. Although this intention-behavior gap is a well-documented phenomenon [52], there is a need for an improved understanding of when and why behaviors deviate from intentions to implement. Specifically, the TPB posits that intentions predict behavior *when one is able to act* [52, 53]. We propose that contextual factors across all CFIR domains might interfere with intentions being translated into action, as shown in Fig. 1. The idea of incorporating the CFIR and TPB builds on work by Mandell and colleagues [31], who suggest that organizational variables are likely to (a) influence attitudes, subjective norms, and self-efficacy and (b) moderate the association between intentions and behavior. Findings from this study similarly suggest that there may be multiple ways in which implementation strategies can enhance TN use. First, implementation strategies may enhance therapists’ intentions to use TNs. Second, implementation strategies that remove barriers between intentions and actions may allow individuals who have strong intentions to follow through with their intended behavior.

Patient needs and resources were identified frequently as contributing to therapists’ intentions to use TNs, as well as interfering with acting on strong intentions. Importantly, therapists’ intentions to use TNs may vary depending on their patient population. Because youth treated by PACTS therapists live in neighborhoods with high rates of poverty and violence [54], this may bring up concerns about using TNs given the context in which young people live. At the same time, working with a child who is experiencing ongoing stressors and/or trauma likely interrupts TN use even for those with strong intentions. Research on the use of TF-CBT for youth with ongoing traumas suggests that there are many cases in which TNs are still appropriate [55]. However, there is a need to adapt TF-CBT for youth with ongoing traumas to ensure that they are being sufficiently supported by therapists and caregivers. This may suggest that a particular focus during training on adapting TF-CBT for use with youth who experience ongoing traumas is warranted [55]. Clinicians also should be encouraged to discuss the appropriateness of TNs during consultation and/or with colleagues trained in TF-CBT.

Although most participants in the present study had strong intentions to use TNs, we identified a number of contextual factors that may influence the development of intentions. For instance, inner setting factors, such as having supervisors and co-workers who use TF-CBT may shape therapists’ perspectives of agency norms. Agency-level implementation strategies focused on addressing normative beliefs, such as bringing awareness to therapists about coworkers who consistently use TNs, may be effective to increase intentions. In addition, other agency-level strategies, such as requiring accountable justification in the electronic health record when clinicians do *not* use TNs, may enhance intentions to use TNs [56].

There was also evidence that inner setting factors interfered with TN use among therapists with high intentions. For example, therapists with high intentions and low TN use reported lower levels of agency support relative to other groups. For therapists delivering interventions in school- or home-based settings, there are likely to be setting-specific implementation challenges. School-based therapists identified concerns about having students return to class dysregulated from TNs. This is consistent with previous research [18, 57] in which participants noted concerns about the fit between TNs and the school setting. Our findings underscore recommendations that TNs warrant particular attention during training and consultation [18]. Furthermore, different settings likely necessitate context-specific implementation strategies following initial training. School settings may need to provide structural changes that allow TN sessions not to occur immediately before a child returns to a stressful situation, such as a test. For clinicians in home-based treatment programs, guidance should be provided on addressing issues of confidentiality (e.g., how to identify a private location to complete TNs). Successful implementation will likely rely on adaptive and appropriately sequenced implementation strategies [58] that are tailored to multiple contextual domains, such as the inner setting.

Finally, it is helpful to consider factors that may differentiate between implementers and non-implementers. Non-implementers were particularly concerned that TNs would worsen clients’ symptoms, despite evidence suggesting that this is not the case. Educational interventions specifically focused on addressing negative beliefs have effectively been used for other exposure-based interventions [59] and may be beneficial to increase self-efficacy and intentions to use TNs, especially with complex cases. It is also helpful to consider the behavior of implementers; participants with high intentions who frequently used TNs were more likely to use a wider array of mediums, such as songs and poems, to deliver TNs. This suggests awareness of flexibility within fidelity [60], or ways in which the protocol can be modified and still meet its core functions. It is possible that implementation strategies such as developing educational materials [61] to be distributed during booster training and consultation could enhance therapists’ understanding of how to flexibly apply TNs. The use of toolkits or workbooks that provide templates for TNs may improve clinician confidence in flexibly implementing manualized interventions [56]. In addition, identifying strategies to enhance the appeal and feasibility of training (i.e., offering CEUs or other incentives) may be particularly relevant for therapists with low intentions and/or low TN usage.

This study has methodological strengths, including its specific focus on TNs rather than on TF-CBT more broadly. This is important given evidence that the strength of intention varies depending on the component of CBT being studied [19]. This variation suggests that underlying beliefs about each component will differ and should be examined separately. Our use of a causal theory and a determinant framework provides unique insight into the implementation of TNs. While the CFIR provides a framework for understanding contextual factors, the TPB contributes an enhanced understanding of how these factors might relate to belief development, motivation, and behavior. Finally, the inclusion of therapists across all PACTS training cohorts allowed us to examine responses for participants who varied in their length of time since training.

This study also had limitations. First, this sample is likely not representative of all therapists conducting TF-CBT. Therapists in this study reported having relatively high intentions to use TNs, which could reflect the fact that they were a self-selected group of therapists who generally favored TF-CBT. Furthermore, this study took place within the outer context of a system that supports TF-CBT implementation, which likely has an impact on therapists’ intentions and beliefs. Another possible explanation for the report of high intentions is that therapists may have reported on intentions to deliver TNs with “ideal” and straightforward cases without accounting for the contextual factors that might affect their intentions with more complex cases. Future research should specifically assess therapists’ understanding of discrepancies between intentions and behavior. Second, the restricted range in TN intentions precluded us from conducting a quantitative assessment of the association between intentions, beliefs, and EBI use, as other studies have done in the past [62]. Third, though therapists reported on their implementation of TNs, we did not objectively measure actual use or fidelity. Studies examining therapist self-report suggest that therapists may overestimate their use of EBIs when therapist self-report is compared to trained observer ratings [63], suggesting that therapists may have used TNs less frequently than they reported. Relatively few constructs were measured quantitatively (e.g., self-efficacy), which is a necessary next step to test the proposed model in Fig. 1. Research will need to identify whether these hypothesized causal pathways differ for individuals with high and low intentions and whether this model applies to interventions other than TNs.

## Conclusions

Results from this study have implications for future implementation research, especially for the delivery of core intervention components that are challenging to deliver. This study helps integrate a causal theory (TPB) with a contextual framework (CFIR). A proposed integration of these approaches appears promising and will need to be quantitatively examined in future work. The stakeholder-generated beliefs reported here can be used to design a quantitative survey questionnaire that can test the causal pathways posited by TPB in a larger sample. Future work should quantitatively test which factors influence belief development and/or moderate the relationship between intentions to use an intervention and actual intervention use. Moreover, there are other theories that aim to understand behavior change during implementation and how it relates to constructs measured by CFIR. Future work should conduct head-to-head comparisons of these theories to evaluate which ones are the most reliable predictors of implementation behavior [36]. This empirical work will not only help identify which theories best predict EBI implementation, but they will also enable researchers to design mechanistically informed implementation strategies for a wide array of interventions.

## Data Availability

The dataset generated and analyzed during the current study is not publicly available due to the highly sensitive nature of interview transcript data. Publication of entire transcripts risk identifying research participants.

## Abbreviations

CBH: Community Behavioral Health
CFIR: Consolidated Framework for Implementation Research
DBHIDS: Department of Behavioral Health and Intellectual disAbility Services
PACTS: Philadelphia Alliance for Child Trauma Services
PTSD: Post-traumatic stress disorder
SAMHSA: Substance Abuse and Mental Health Services Administration
TPB: Theory of Planned Behavior
TF-CBT: Trauma-focused cognitive behavioral therapy
TN: Trauma narrative

## Footnotes

1 Given that TPB calls for focusing on specific behaviors, the interviews aimed to focus on TNs. However, many participants also discussed the broader package of TF-CBT even when questions were focused on TNs. Although analyses focused primarily on statements related to TNs, some themes emerged related to TF-CBT more broadly.
